# Ni_2_P Nanoparticle-Inserted Porous Layered NiO Hetero-Structured Nanosheets as a Durable Catalyst for the Electro-Oxidation of Urea

**DOI:** 10.3390/nano12203633

**Published:** 2022-10-17

**Authors:** Kun Ma, Hui Wang, Palanisamy Kannan, Palaniappan Subramanian

**Affiliations:** 1 Department of Internet, Jiaxing Vocational Technical College, Jiaxing 314001, China; 2 College of Biological, Chemical Sciences and Engineering, Jiaxing University, Jiaxing 314001, China; 3 New Technologies-Research Center, University of West Bohemia, 30100 Plzeň, Czech Republic

**Keywords:** hetero-nanostructures, Ni_2_P@NiO nanosheets, electro-oxidation of urea, wastewater, environmental safety

## Abstract

The electro-oxidation of urea (EOU) is a remarkable but challenging sustainable technology, which largely needs a reduced electro-chemical potential, that demonstrates the ability to remove a notable harmful material from wastewater and/or transform the excretory product of humans into treasure. In this work, an Ni_2_P-nanoparticle-integrated porous nickel oxide (NiO) hetero-structured nanosheet (Ni_2_P@NiO/NiF) catalyst was synthesized through in situ acid etching and a gas-phase phosphating process. The as-synthesized Ni_2_P@NiO/NiF catalyst sample was then used to enhance the electro-oxidation reaction of urea with a higher urea oxidation response (50 mA cm^−2^ at 1.31 V vs. RHE) and low onset oxidation potential (1.31 V). The enhanced activity of the Ni_2_P@NiO/NiF catalyst was mainly attributed to effective electron transport after Ni_2_P nanoparticle insertion through a substantial improvement in active sites due to a larger electrochemical surface area, and a faster diffusion of ions occurred via the interactive sites at the interface of Ni_2_P and NiO; thus, the structural reliability was retained, which was further evidenced by the low charge transfer resistance. Further, the Ni_2_P nanoparticle insertion process into the NiO hetero-structured nanosheets effectively enabled a synergetic effect when compared to the counter of the Ni_2_P/NiF and NiO/NiF catalysts. Finally, we demonstrate that the as-synthesized Ni_2_P@NiO/NiF catalyst could be a promising electrode for the EOU in urea-rich wastewater and human urine samples for environmental safety management. Overall, the Ni_2_P@NiO/NiF catalyst electrode combines the advantages of the Ni_2_P catalyst, NiO nanosheet network, and NiF current collector for enhanced EOU performance, which is highly valuable in catalyst development for environmental safety applications.

## 1. Introduction

Urea has been recognized as an active H_2_ transporter and CO_2_ storage system for constant energy sources owing to its intrinsic features: mainly its high energy density (16.9 MJ L^−1^, 6.67 wt.% of hydrogen), nonflammability, extensive accessibility as a major component in human urine, natural abundance, eco-friendliness, simplicity of transport, and low storage cost [1,2]. Urea-rich wastewater has been increasing environmental contamination owing to industrial plant effluents [3,4]. It is well-known that the manufacturing effluent of urea might yield large quantities of urea-rich wastewater when compared to the urine discharge by human and animals. Notably, 0.75 kg urea-rich waste effluents is discharged through the manufacturing of 1 kg urea with nearly 1 wt.% content of urea. Although urea is not a poisonous chemical, its degradation leads to severe issues for eco-systems [5].

Urea-rich wastewater is a conventional nitrogen-comprising wastewater; day-by-day, urea is merged with composts and feed-covered substances. It is mainly unsafe to the environment when urea is distributed via the air and followed by a typical modification to hazardous substances, and medicinal disorder is also produced when drinking water contaminated by urea. Henceforth, the progress of a sustainable approach to the elimination of urea-rich wastewater is extremely crucial. However, from the numerous available approaches, electro-oxidation is one of the favorable techniques for urea oxidation owing to its prominent characteristics [6,7]. The electro-oxidation of urea produces a valuable energy product (H_2_), a nontoxic product (N_2_), and CO_2_; although if the urea is a run-off from natural bases, the production of CO_2_ is a portion of the biological carbon cycle [8]. The electro-oxidation of urea has received growing consideration in the electrochemical method of the ecofriendly catalysis of urea–wastewater management [6,9,10,11,12,13,14,15,16,17,18]. Hydrogen can be generated by the dissociation of urea, and it can also be immediately served to fuel cell systems to produce electricity [19,20]. In addition, urea oxidation is a favorable alternate reaction for water oxidation when used for the generation of hydrogen [21,22,23,24,25]. However, the EOU can suffer with sluggish kinetics due to a six-electron transfer procedure. Thus, a high-performance and low-cost catalyst for the EOU is significantly crucial to speed up the slow kinetic reaction [26,27]. As a significant electrochemical reaction for energy transformation and catalysis, several studies have been developed regarding nanostructured catalysts for the electro-oxidation of urea [28]. For instance, several studies have demonstrated that noble metal catalytic nanostructured materials such as Ru, Pt-, and Pd-based nanocomposites when used as anode catalysts exhibited a higher catalytic activity of the EOU [29,30,31,32]; however, their industrial use is restricted by cost-value and shortage. Hence, continuous studies are being undertaken to develop inexpensive, earth-abundant and low-cost non-noble metallic electrocatalyst materials for the EOU.

Among the available earth-abundant catalyst materials for the EOU, nickel (Ni)-based catalysts have fascinating widespread benefits, together with nickel hydroxide, nickel phosphide, nickel oxide, and so on [33,34,35]. Particularly, nickel hydroxide has revealed an outstanding catalytic activity that is comparable to noble metals based on the electro-oxidation of urea. Notably, nickel hydroxide has shown two polymorphs: α-Ni(OH)_2_ and β-Ni(OH)_2_. During the preoxidation process before the EOU or the oxygen evolution reaction (α(β)-Ni(OH)_2_ + OH^−^ ↔ γ(β)-NiOOH + H_2_O + e^−^), NiOOH with a higher valence of Ni^3+^ can be produced in both phases, which has been known as a dynamic surface site in electrochemical reactions [36,37]. However, the inadequate active sites and lower electronic carriage ability still limit their potential usage in nickel-based fuel cell applications. To solve the above issues, inserting active nanoparticles has been demonstrated to be an efficient technique to alter the electronic structure of catalysts.

For instance, He et al. grew NiFe_2_O_4_ nanoparticles on NiFe(OH)x nanosheets through a laser ablation approach. Notably, the NiFe(OH)x/NiFe_2_O_4_ hybrid catalysts exhibited an electrocatalytic performance toward the EOU (with a potential of 1.343 V at 10 mA cm^−2^ and a Tafel slope of 19.9 mV dec^−1^) [38]. Liu et al. synthesized Ni/Mo_2_C@CN nanocomposites composed of porous carbon shell and a hetero-junction Ni/Mo_2_C core through an in situ self-confinement approach. The combined catalytic activity of Ni and Mo_2_C had increased activity of both urea oxidation and hydrogen evolution reactions simultaneously by applying 1.51 V to reach 10 mA/cm^2^ of complete urea oxidation on an Ni/Mo_2_C@CN catalyst [39]. On the other side, Zhang and coworkers prepared a sequence of Ni-Co on carbon cloth (Ni-Co@CC) using a hydrothermal process and then treated it with a phosphidation reaction. The morphology of the nanomaterials and electronic configuration were controlled by the content of the Ni and Co precursors. Notably, Ni-Co@CC showed a low operational potential in the urea-containing electrolyte; thus, Ni-Co@CC exhibited the greatest EOU catalytic display, with a potential of 1.362 V at η_10_ in 1.0 M KOH + 0.33 M urea [40]. Inspired by the above reports, we synthesized Ni_2_P nanoparticle-inserted NiO nanosheets on an NiF substrate (Ni_2_P@NiO/NiF) as a bifunctional catalyst using in situ acid etching and a phosphating method. Thereafter, Ni_2_P@NiO/NiF can be used for the enhanced electro-oxidation of urea; the EOU current response of 10 mA cm^−2^ was achieved at 1.338 V, which is superior to other catalysts, NiO, Ni_2_P, and commercially available RuO_2_ nanoparticles.

## 2. Experimental Section

### 2.1. Chemicals and Reagents

Sodium hypophosphite hydrate (NaH_2_PO_2_·H_2_O, 99.0%) and HCl (37.0%) were obtained from Sinopharm Chemical Reagent Co., Ltd., Shanghai, China. The working electrode material, nickel foam (thickness 1.5 mm, porosity greater than 95–98%, density > 0.1 g/cm^3^) was received from Shanxi LZY Battery Materials Co., Ltd., Shanxi, China.

### 2.2. Synthesis of the NiO/NiF Catalyst Sample

Initially, nickel foam (NiF, 2 × 2 cm) was dipped into 1.0 M HCl, then ultrasonicated for 15 min to clean surface oxides and thereafter, washed with Millipore water for few times. Pretreated NiF was kept in 20.0 mL of 3.6 mM HCl and treated at 100 °C for 20 h in Teflon-lined stainless steel autoclave vessel; afterwards, it was rinsed with Millipore water a few times and immediately vacuum-dried at 60 °C. The above synthesized substrate was designated as Ni(OH)_2_/NiF. For comparison purposes, NiO/NiF was fabricated by Ni(OH)_2_/NiF electrode treated at 350 °C for 2 h.

### 2.3. Synthesis of the Ni_2_P@NiO/NiF Catalyst Sample

The as-fabricated NiO/NiF substrate was placed in a porcelain boat and carefully kept inside a tubular furnace; simultaneously, 0.5 mmol of NaH_2_PO_2_ was taken in another porcelain boat, and it was placed in an upstream position. Thereafter, the tubular furnace was blushed with nitrogen gas for 10 min and heated to 350 °C at the ramp of 5 °C/min. The NiO/NiF substrates with various phosphating times (20, 40, and 60 min) were prepared at 350 °C. The phosphatized substrates were denoted as Ni_2_P@NiO/NiF-N, where “*n*” indicates the different phosphating periods. The mass loading of electrocatalysts on Ni foam substrate was about 36.53, 36.92, and 38.31 mg cm^−2^ for Ni_2_P/NiF, NiO/NiF, and Ni_2_P@NiO/NiF, respectively.

### 2.4. Characterizations of the As-Synthesized Nanostructures

Size and surface morphology of as-synthesized Ni_2_P@NiO/NiF nanostructures were examined using JEOL JSM-7100F scanning electron microscope, JEM-2000 FX JEOL transmission electron microscope functioning at 200 kV with STEM, and SAED techniques. XRD was performed using Shimadzu XD-3A equipment fitted with Cu-Kα radiation source (k = 0.15418 nm) and worked at 30 mA and 40 kV. XPS was recorded using VG Escalab-210 equipment with Mg-300-W X-ray supply. Reference value was selected for adjusting influential deviations mainly from binding energy of C 1 s, i.e., 284.8 eV, and total number of scans was measured with a difference of 0.1 eV.

### 2.5. Electrochemical Measurements

Electrochemical setup connected to a CHI 760 electrochemical workstation (CH Instruments, Bee Cave, TX, USA) was used to investigate electro-oxidation of urea. The Hg/HgO and graphite rod were used as reference and counter electrodes, respectively. For comparison analysis, commercial RuO_2_ nanoparticle-modified NiF electrode was fabricated by dissolving 8.0 mg of the catalyst sample, 1.0 mL of PTFE polymer binder, and 1.0 mg of acetylene black in 150 μL of isopropyl alcohol treated in ultrasonic bath for 15 min to produce homogenous ink. In addition, 1.0 M KOH + 0.33 M urea and 1.0 M KOH alone were used as electrolytes for systematic study. All the measured electrode potentials were converted to reversible electrode (RHE) potential according to the formula:

E_RHE_ = E_Hg/HgO_ + 0.059 pH + 0.098 (1)

## 3. Results and Discussion

### 3.1. Synthesis of the Ni_2_P@NiO Nanostructures

**Scheme 1:**
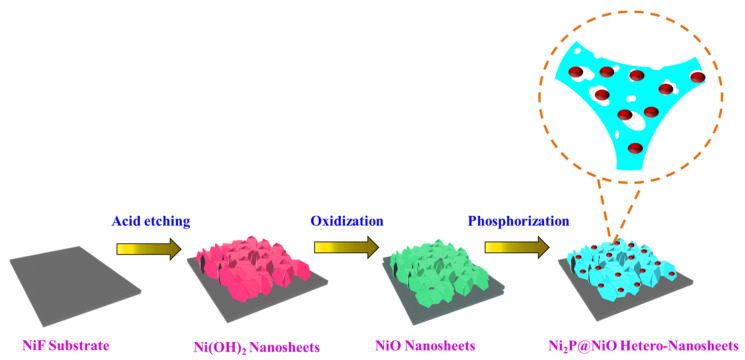
Pictorial representation showing the growth of porous hetero-structured Ni_2_P@NiO nanosheets on the surface of NiF substrate.

**Figure 1:**
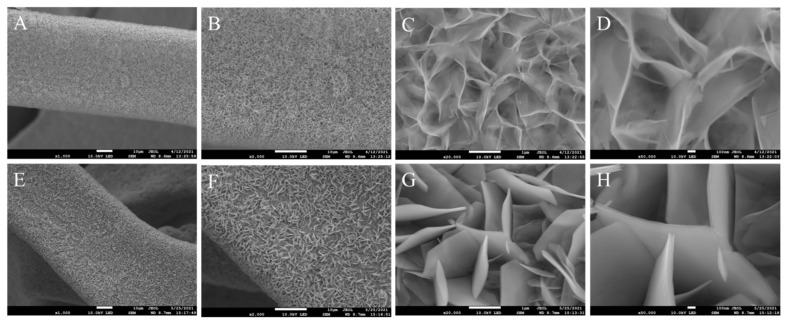
Low- to high-magnified SEM images of Ni(OH)_2_/NiF-40 (**A**–**D**) and NiO/NiF (**E**–**H**), which showing the porous layered nanosheets network structures. Scale Bars: 10 μm (**A**,**B**,**E**,**F**); 1.0 μm (**C**,**G**), and 100 nm (**D**,**H**).

The fabrication procedure of the hetero-structured Ni_2_P nanoparticle-inserted NiO nanosheets on an NiF surface is demonstrated in Scheme 1. First, the etching of NiF was carried out through a weak acid treatment based on the given chemical reactions: Ni + 2H^+^ ↔ Ni^2+^ + H_2_ (i); and Ni^2+^ + 2OH^−^ ↔ Ni(OH)_2_ (ii); after the completion of the reaction, the nanosheets of Ni(OH)_2_ were developed on the NiF surface. The construction of the identical 3D networked nanosheets of Ni(OH)_2_ were achieved using the NiF substrate hydrothermally treated with HCl (Figure 1A–D). The fairly even, upright, external, compact and united, thin, solid, wall-like 3D formation of the nanosheet layers on the surface of the NiF substrate were observed, which was promising for the exploitation of active materials (Figure 1A–D) for an EOU reaction. The solid wall-like nanosheets were composed of numerous discrete nano-walls with the lengths of 1–2 μm and the thickness of 30–50 nm (Figure 1D). Further, the acquired 3D networked Ni(OH)_2_ solid wall-like nanosheets were treated at 350 °C for 2 h, which ensued in the decomposition of Ni(OH)_2_ to produce NiO/NiF. Figure 1E–H reveals the unique robust and stable wall-like 3D nanosheet-like morphologies of the NiO/NiF retained after calcination, demonstrating the robustness of the 3D networked wall-like nanosheet structure at a higher temperature. Next, the above NiO/NiF substrate was phosphated by NaH_2_PO_2_ at different periods. The obtained Ni_2_P@NiO/NiF-*x* samples restored the similar wall-like nanosheet morphology (see later).

**Figure 2:**
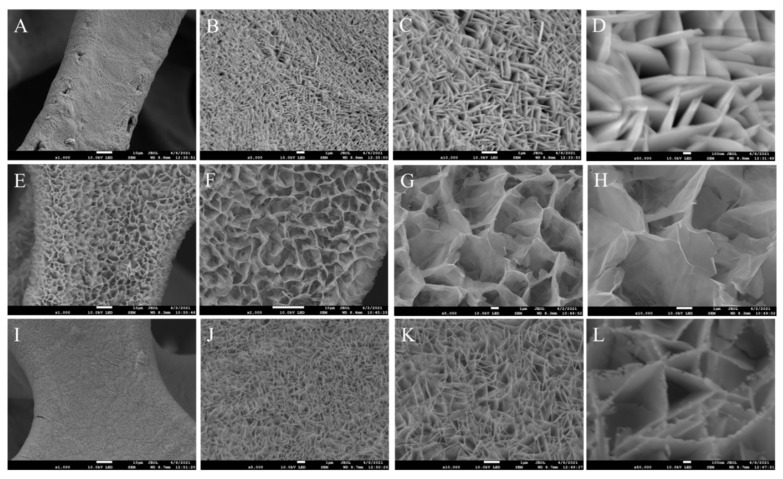
Low- to high-resolution SEM images showing formation of Ni_2_P nanoparticles on NiO/NiF wall-like networked nanosheet surface at different time intervals. (i) 20 min (images **A**–**D**), (ii) 40 min images (**E**–**H**), and (iii) 60 min (images **I**–**L**). Scale Bars: 10 μm (**A**,**E**,**F**,**I**), 1 μm (**B**,**C**,**G**,**J**,**K**), and 100 nm (**D**,**H**,**L**).

**Figure 3:**
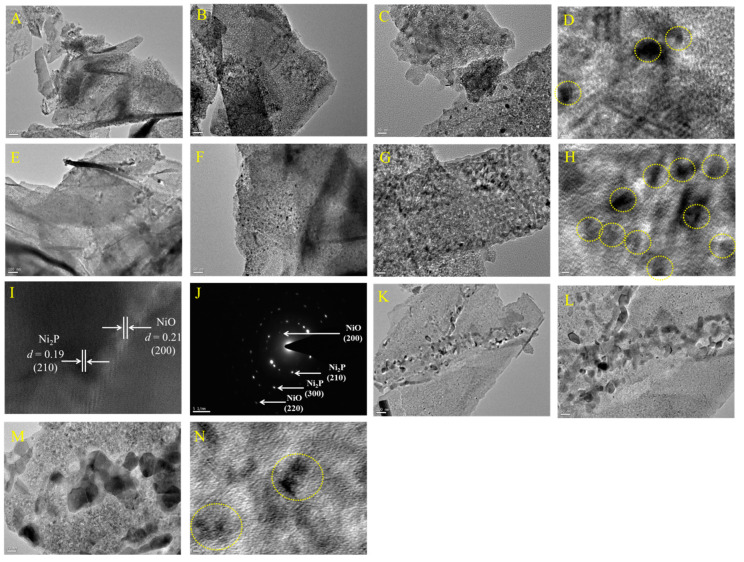
Low- to high-resolution TEM images showing the insertion of Ni_2_P nanoparticles on the surface of NiO/NiF wall-like networked nanosheets at different time intervals. (i) 20 min (images A–D), (ii) 40 min images (E–I), corresponding selected area diffraction pattern (SAED; **J**), and (iii) 60 min (images **K**–**N**). **Scale Bars:** 100 nm (images **A**,**E**,**K**); 50 nm (images **B**,**F**,**L**); 20 nm (images **C**,**G**,**M**); and 2 nm (images **D**,**H**,**I**,**N**). Scale bar for SAED (**J**): 5 nm.

A comprehensive study was carried out for the formation of various Ni_2_P@NiO/NiF-*x* (*x* is 20, 40, and 60 min) catalysts using SEM and TEM microscopes. Several layers of highly uniform 3D interconnected wall-like nanosheets formed on the NiF surface; on further examination, the insertion of time-dependent Ni_2_P nanoparticles is illustrated, as shown in Figure 2A–L. It is important to mention here that only a few Ni_2_P nanoparticles (20 min growth time) were formed on the surface of the smooth wall-like NiO nanosheets (see Figure 2A–D); whereas as the growth time increased (40 min), the toughness and roughness of the wall-like NiO nanosheets were slightly altered due to the formation of a large amount of Ni_2_P nanoparticles (see Figure 2E–H). However, the Ni_2_P nanoparticles tended to aggregate on the surface of the wall-like NiO nanosheets due to an extended reaction time (60 min), which resulted in more roughness and the uneven nature of the NiO nanosheets as well as the formation of irregular-sized Ni_2_P nanoparticles on the surface of the NiO nanosheets (Figure 2I–L). The formation of Ni_2_P nanoparticles on the surface of the NiO wall-like networked nanosheets was further characterized using TEM measurements. The porous nanostructure was observable in all of the TEM images of Ni_2_P@NiO/NiF (Figure 3A–N); however, the insertion of the Ni_2_P nanoparticles varied in the nanosheets, depending on the phosphating process reaction time. Low- to high-magnification TEM images revealed that a 20 min growth time resulted in only a few Ni_2_P nanoparticles with a size range of 5 ± 1 nm, randomly inserted on the surface of the NiO wall-like networked nanosheets, which are shown in Figure 3A–D (yellow circled marks in Figure 3D). Notably, the uniform distribution of a large amount of the uniform Ni_2_P nanoparticles with a size range of 4 ± 1 nm were inserted on the surface of the NiO wall-like networked nanosheets upon the 40 min reaction time, indicating the significance of growth time. The TEM images revealed the existence of several surface pores occupied by numerous nanoparticles (Figure 3E–H). Notably, a well-poised lattice pattern with interplanar spaces of ca. ~0.21 and ~0.19 nm were observed in the HRTEM (Figure 3I), which is ascribed to the existence of NiO (200 plane) and Ni_2_P (210 plane), respectively, demonstrating the existence of the NiO nanosheets and the Ni_2_P nanoparticles embedded within their pores [41]. Further, a selected area of the electron diffraction (SAED) image of the Ni_2_P@NiO/NiF catalyst revealed the presence of the (200) and (220) planes of the NiO nanosheets along with the (210) and (300) planes of the Ni_2_P nanoparticles (Figure 3J), indicating that both hetero-elements were found in the sample. The insertion of the Ni_2_P nanoparticles continued with an increasing phosphidation reaction time (60 min) that resulted in uneven and aggregated nanoparticles with a size of 20 ± 4 nm on the NiO wall-like networked nanosheet structures (Figure 3K–N), implying that the 40 min phosphating process was optimal to obtain an optimal catalyst for further studies.

**Figure 4:**
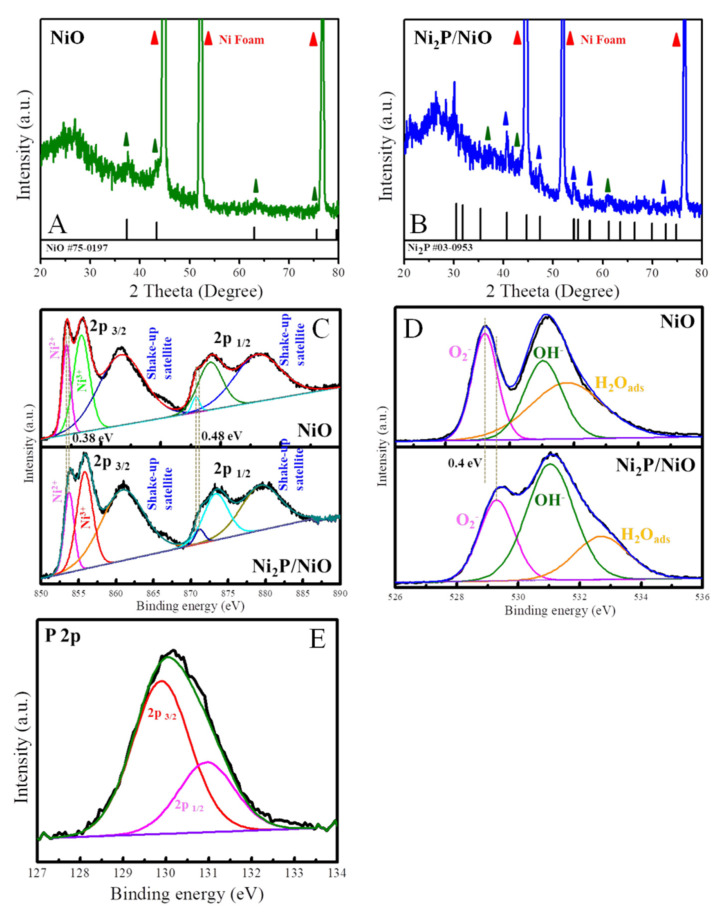
XRD pattern profiles of NiO/NiF (**A**) and Ni_2_P@NiO/NiF (**B**) catalyst samples. XPS spectra obtained for Ni 2p of Ni_2_P@NiO/NiF and NiO/NiF (**C**); O 1s of Ni_2_P@NiO/NiF and NiO/NiF (**D**); and P 2p of Ni_2_P@NiO/NiF (**E**) catalyst samples.

The Ni_2_P nanoparticle formation on the NiO/NiF wall-like networked nanosheets can be ascribed to the Kirkendall effect [42,43]. The NaH_2_PO_2_ precursor decomposition occurred at 273 °C, produced the gas phases of PH_3_, and further decomposed to H_2_ that, in turn, reduced the Ni^2+^ ions to a zerovalent Ni state, which permitted them to diffuse in an outward direction during the phosphating process. At the same time, P cumulated inside and reacted with Ni^2+^ to produce the Ni_2_P nanoparticles. The diffusion level of Ni was rapid in contrast to P, and it moved into reverse paths due to the Kirkendall process. The variance in the migration rate initiated the generation of numerous pores on the NiO nanosheets, which allowed the Ni_2_P nanoparticles. This Kirkendall effect becomes more significant with respect to the phosphating time (see Figure 3). We further performed an EDX analysis of the Ni_2_P@NiO/NiF catalyst sample, which clearly confirmed the existence of Ni, O, and P components on its surface (Figure S1A). In addition, the STEM and EELS elemental mapping evidenced the presence of Ni, O, and P in the NiO/NiF catalyst sample with a significant coverage of the Ni_2_P nanoparticles in the inner pores and surface walls (Figure S1B–E). The hetero-nanostructure was then characterized using XRD pattern analysis. As shown in Figure 4A, the diffraction patterns of the as-prepared NiO wall-like networked nanosheets fitted well with that of the standard NiO (JCPDS No. 75-0197) phases. Particularly, the four characteristic peaks at 37.4°, 43.6°, 63.6° and 74.4° corresponded to the (111), (200), (220), and (222) diffraction planes, respectively [44,45]. Notably, no other peaks were detected in the catalyst sample, demonstrating the purity of the product. In addition, no peaks from the Ni substrate were detected, indicating that the NiO nanosheets consistently grew up on the surface of the Ni foam substrate. The insertion of the Ni_2_P nanoparticles on the surface of the NiO wall-like nanosheets resulted in five new peaks at 40.8°, 47.9°, 54.6°, 55.2°, and 73.8° accredited to the (111), (210), (300), (211), and (311) planes of Ni_2_P (JCPDS No. 03–0953) (Figure 4B) [46,47]. The intensity of the NiO diffraction peaks was slightly diminished due to the phosphidation process. In addition, the evolution of the Ni_2_P peaks was predominant at 40 min phosphating time (Figure S2), which could be potentially used for an electro-oxidation reaction (see later). The XPS analysis was performed to examine the chemical state and surface composition of the Ni(OH)_2_/NiF, NiO/NiF, and Ni_2_P@NiO/NiF-40 catalyst samples. The Ni 2p XPS of the Ni(OH)_2_/NiF nanosheet structure was deconvoluted into two doublets at 855.4 and 872.9 eV, and its shake-up satellites potentially evidenced the existence of Ni^2+^ (Figure S3A). Further, the deconvoluted O 1 s XPS of the Ni(OH)_2_/NiF sample displayed three peaks in the position of 529.4, 530.8 and 531.8 eV (Figure S3B) that can be ascribed to the presence of the O^2−^, OH^−^, and H_2_O_ads_ species, respectively. Notably, the existence of the OH^−^ and H_2_O_ab_ peaks demonstrated the growth of the Ni(OH)_2_ nanosheets over the NiF during the process of the acid-etching [48,49]. Next, the Ni 2p XPS of NiO/NiF displayed two peaks at 853.1 eV and 871.2 eV that were attributed to Ni 2p_3/2_ and Ni 2p_1/2_, respectively (Figure 4C), evidencing the occurrence of Ni^2+^. However, the peaks at 855.6 eV and 872.5 eV corresponded to Ni^3+^ and confirmed the presence of NiOOH on the surface of the NiF substrate via the oxidation process [50]. Notably, the Ni 2p XPS of Ni_2_P@NiO/NiF deconvoluted into two doublets along with their shake-up satellites peaks located at 853.5 eV and 871.7 eV, originating at the Ni^2+^ component and shifted to a binding energy range by 0.38 eV and 0.48 eV that related to the NiO/NiF catalyst (Figure 4D). The above observed shift was mainly ascribed to the binding of the O, P, and Ni elements on Ni_2_P@NiO, which clearly confirmed the insertion of the Ni_2_P nanoparticles on the NiO nanosheet structure [51,52]. The O 1 s XPS of the Ni_2_P@NiO/NiF-40 and NiO/NiF samples are displayed in Figure 4D. The O 1 s deconvoluted into three components at 528.9, 530.7, and 531.5 eV, corresponding to the lattice oxygen, the hydroxyl component, and the structurally adsorbed water, respectively. The O^2–^ position moved from 528.9 eV to 529.3 eV due to the phosphidation reaction, which was associated with the reorganization of the charges on the Ni_2_P@NiO interface and made the connection between the Ni_2_P and NiO [53]. The P 2p region of the Ni_2_P@NiO/NiF-40 sample showed two peaks at 129.8 eV and 131.0 eV, which were mainly associated with 2p_3/2_ and 2p_1/2_, respectively (Figure 4E) [53]. This result distinctly demonstrates the presence of Ni-P-O chemical bonding and evidences the insertion of the Ni_2_P component in the Ni_2_P@NiO/NiF-40 sample.

### 3.2. Electro-Oxidation of Urea (EOU) at the Ni Catalyst-Based Electrodes

**Figure 5:**
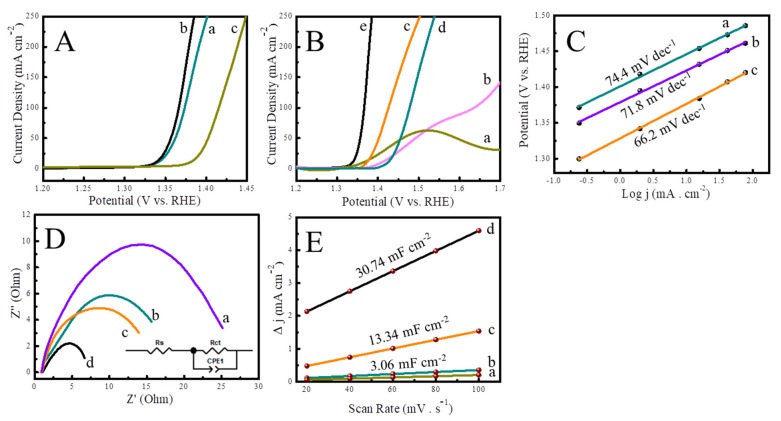
(**A**) LSVs obtained for Ni_2_P@NiO/NiF-20 (curve a), Ni_2_P@NiO/NiF-40 (curve b), and Ni_2_P@NiO/NiF-60 (curve c) catalyst electrodes in 1.0 M KOH + 0.33 M urea at a scan rate of 5 mV s^−1^. (**B**) LSVs obtained for unmodified NiF (curve a), RuO_2_ NPs/NiF (curve b), NiO/NiF (curve c), Ni_2_P NPs/NiF (curve d), and Ni_2_P@NiO/NiF-40 (curve e) catalyst electrodes in 1.0 M KOH + 0.33 M urea at a scan rate of 5 mV s^−1^. (**C**) Tafel plots obtained for Ni_2_P NPs/NiF (line a), NiO/NiF (line b), and Ni_2_P@NiO/NiF (curve c). (**D**) EIS and corresponding electrochemical double-layer capacitance (**E**) obtained for unmodified NiF (curve a), Ni_2_P NPs/NiF (curve b), NiO NPs/NiF (curve c), and Ni_2_P@NiO/NiF-40 (curve d) catalyst electrodes in 1.0 M KOH + 0.33 M urea solution.

The three different EOU, as-synthesized catalysts, Ni_2_P@NiO/NiF-20, Ni_2_P@NiO/NiF-40, and Ni_2_P@NiO/NiF-60 were used to examine their catalytic activities using a linear sweep voltammetry (LSV) method in nitrogen-saturated 1.0 M KOH + 0.33 M urea at a scan rate of 5 mV s^−1^. As can be seen in Figure 5A, there was no oxidation peak; however, the onset potential of the EOU in the presence of 0.33 M urea is consistent with the onset potential of a urea oxidation peak, proving that the oxidized catalysts were electro-active substances toward the electro-oxidation reaction of urea [1,54]. In particular, Ni_2_P@NiO/NiF-20 displayed distinguished EOU at an onset potential of 1.34 V, and it reached 50 mA cm^−2^ at a potential of 1.36 V (Figure 5A; curve a). Next, the Ni_2_P@NiO/NiF-40 catalyst electrode significantly displayed a much better EOU profile with a comparatively lower onset potential (1.31 V), and the above catalyst electrode reached 50 mA cm^−2^ at a potential of 1.35 V (Figure 5A; curve b). Lastly, the Ni_2_P@NiO/NiF-60 catalyst electrode showed a diminished EOU profile with an onset of 1.37 V and reached 50 mA cm^−2^ at a potential of 1.40 V (Figure 5A; curve c). We have compared the EOU catalytic activities using other catalysts such as Ni_2_P nanoparticles, NiO nanosheets, and commercial RuO_2_ nanoparticle-modified NiF and unmodified NiF electrodes under identical experimental conditions. As shown in Figure 5B, the unmodified NiF electrode displayed a low catalytic EOU activity (Figure 5B; curve a), which indicated that the NiF substrate alone was not sufficient to oxidize urea. Next, the RuO_2_ nanoparticle-modified electrode (RuO_2_ NPs/NiF) displayed a slightly improved EOU reaction with the onset potential of 1.35 V, and this RuO_2_ NPs/NiF electrode reached 50 mA cm^−2^ at a potential of 1.45 V (Figure 5B; curve b). On the other side, the catalysts such as the NiO nanosheets (NiO NS/NiF; Figure 5B; curve c) and the Ni_2_P nanoparticle-modified electrodes (Ni_2_P NPs/NiF; Figure 5B; curve d) showed an almost identical EOU profile, i.e., the onset oxidation potentials were at 1.34 V and 1.38 V, and both the electrodes reached 50 mA cm^−2^ at a potential of 1.40 V and 1.45 V, respectively. Interestingly, the Ni_2_P@NiO/NiF-40 catalyst electrode showed a significantly higher electro-oxidation current density (Figure 5B; curve e: 50 mA cm^−2^ at 1.34 V) with an onset oxidation potential of 1.31 V (vide supra) at a scan rate of 5 mV s^−1^.

**Scheme 2:**
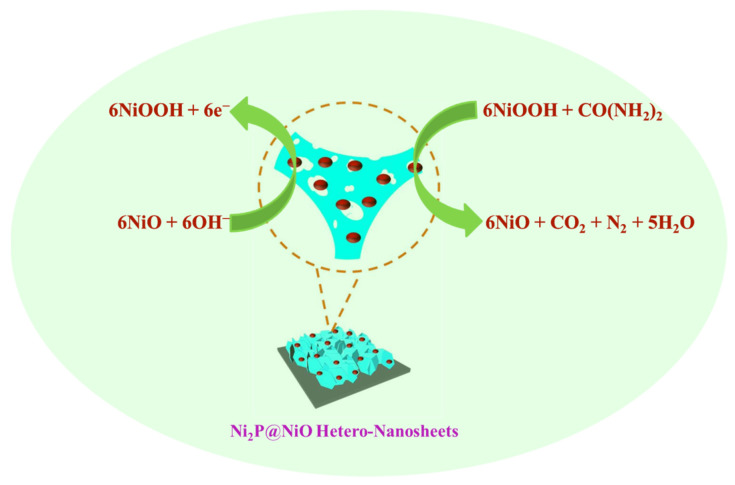
Graphic illustration showing that the oxidation mechanism of urea occurring on the surface of the porous hetero-nanostructured Ni_2_P@NiO/NiF nanosheets.

The enhanced EOU activity is mainly ascribed to the following reasons: (i) Ni_2_P@NiO/NiF-40 showed a good electrical conduction and highly stable mechanical property, which enables the rapid flow of electrons; (ii) the uniform Ni_2_P@NiO/NiF-40 wall-like networked nanosheets were interconnected to each other and composed of several mesopores, which not only displayed electrically networked dense active sites but also enabled the diffusion of electrolyte and urea molecules for an efficient and sustained oxidation reaction; (iii) the Ni_2_P@NiO/NiF-40 catalyst showed a synergism between Ni_2_P nanoparticles and NiO nanosheets, which improved the electrocatalytic reaction kinetics and thus, resulted in higher catalytic current responses toward the electro-oxidation of urea. During the EOU process, the NiO species was electrochemically transformed to NiOOH, and subsequently, the active species of NiOOH catalyzed the urea oxidation, and simultaneously, NiO was regenerated. This cycling generation and reduction in the NiOOH process was considered as a key step in the mechanism of urea oxidation [55]. Thus, the electro-oxidation mechanism of urea can be derived based on Equations (1) and (2) below as well as Scheme 2 because Ni(OH)_2_ was oxidized to the active oxidation state of Ni^3+^ in NiOOH, and subsequently, the adsorbed urea molecules approached the NiOOH surface to form end-products such as N_2_, CO_2_, and H_2_O, and simultaneously, NiO was regenerated on the electrode [56,57]. The Ni_2_P NPs catalyst acted as a co-catalyst (synergy) to boost the urea oxidation process in the same electrochemical oxidation reaction.

6NiO + 6OH^−^→ 6NiOOH + 6e^−^ (2)

6NiOOH + CO(NH_2_)_2_ → 6NiO + CO_2_ + N_2_ + 5H_2_O (3)

Here, it is important to mention that without urea in the 1.0 M KOH solution, an oxidation peak appeared at about 1.36 V vs. RHE, which is attributed to the oxidation of the Ni catalyst, and further scanning resulted in only the OER process that occurred at 1.7 V (Figure S4; curve a) and clearly revealed the EOU occurrence only in the existence of 0.33 M urea in 1.0 M KOH (Figure S4; curve b). In addition, mass activity-based EOU profiles were obtained, which clearly revealed that the Ni_2_P@NiO/NiF catalyst electrode showed a considerably higher electro-oxidation response toward urea (Figure S5). A Tafel slope was acquired to assess the electro-catalytic kinetics of the EOU [58]. A Tafel slope is plotted by using the LSV method to study the EOU via plotting the electrode potential vs. the logarithm of the absolute current density. The Tafel value from the plot (mV dec^−1^) revealed the exact EOU mechanism; the lower Tafel value evidenced that the charge transfer reaction between the analyte/electrolyte and electrode was faster. As shown in Figure 5C, the Tafel slope values of 74.4, 71.8, and 66.2 mV dec^−1^ were obtained for the Ni_2_P NPs/NiF (line a), NiO/NiF (line b), and Ni_2_P@NiO/NiF (line c) electrodes, respectively. The Ni_2_P@NiO/NiF catalyst displayed a lower Tafel slope value than the other catalyst-modified electrodes (Ni_2_P/NiF, and NiO-NiF) demonstrating rapid kinetics of the urea oxidation response by scanning more positive over-potential side. The EOU kinetics of the catalysts was further examined using the EIS technique, which is assembled using an equivalent circuit model comprising three key components: R_ct_ (charge transfer resistance), R_s_ (bulk solution resistance), and CPE (constant phase element) [9,59]. The fitted result (Figure 5D) shows that the Ni_2_P@NiO/NiF nanosheets (6.8 Ω; curve d) showed the lowest charge transfer impedance when compared with the unmodified NiF (25.2 Ω; curve a), Ni_2_P NPs/NiF (16.5 Ω; curve b), and NiO NS/NiF (14.1 Ω; curve c) catalyst-modified electrodes, suggesting a greater electron transfer rate and improved kinetics for the EOU reaction. The lower charge transfer resistance of the Ni_2_P@NiO/NiF nanosheets is mainly attributed to the introduction of the Ni2P nanoparticles into the porous NiO nanosheets, which exposed a large active surface area and enhanced the electrocatalytic activity during the EOU reaction. Next, to study the EOU catalytic activity, the ECSA of all the catalyst-modified electrodes was evaluated using double-layer capacitance (C_dl_) because of its linear relationship with ECSA [60]. The C_dl_ was attained using CV responses at various scan rates (Figure S6), which can be used to calculate the effective electro-active surface area of catalysts [61]. Plotting Δi = 1/2 (i_anodic_ − i_cathodic_) at −0.04 V was mainly associated with applied scan rates, and the respective slope revealed the C_dl_ value [62], as shown in Figure 5E. The Ni_2_P@NiO/NiF nanosheet-modified electrode exhibited a larger C_dl_ value (30.74 mF cm^−2^) than the other catalyst-modified electrodes such as the unmodified NiF (1.96 mF cm^−2^), Ni_2_P NPs/NiF (3.06 mF cm^−2^), and NiO NS/NiF (13.34 mF cm^−2^), indicating that the Ni_2_P@NiO/NiF nanosheet-modified electrode had the highest ECSA and excellent intrinsic catalytic activity for enhanced EOU.

### 3.3. EOU Stability and Wastewater Analyses at the Ni Catalyst-Based Electrodes

**Figure 6:**
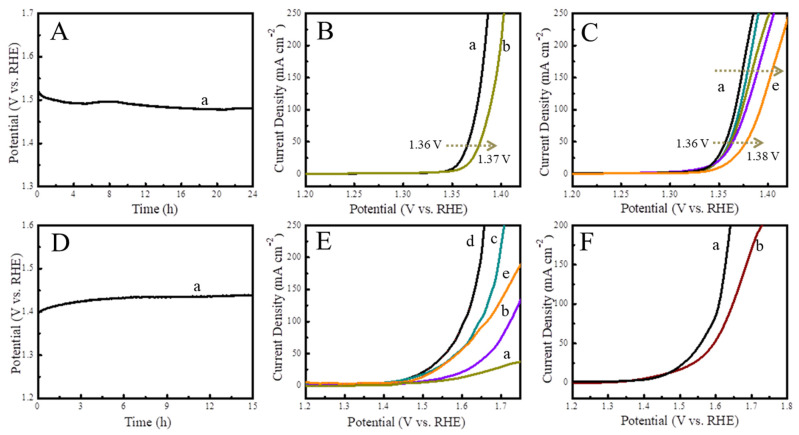
(**A**) Chronopotentiometry curve obtained for Ni_2_P@NiO/NiF-40 (curve a) catalyst electrode in 1.0 M KOH + 0.33 M urea at applied current density of 50 mA cm^−2^. (**B**) LSVs obtained for Ni_2_P@NiO/NiF-40 (initial: curve a, and after 24 h: curve b) catalyst electrode in 1.0 M KOH + 0.33 M urea at a scan rate of 5 mV s^−1^. (**C**) LSVs obtained for Ni_2_P@NiO/NiF-40 catalyst electrode at different days (curves a–e: fresh, 1, 3, 5, and 7 days) in 1.0 M KOH + 0.33 M urea at a scan rate of 5 mV s^−1^. (**D**) Chronopotentiometry curve obtained for Ni_2_P@NiO/NiF-40 (curve a) catalyst electrode (7 days stored) in 1.0 M KOH + 0.33 M urea at applied current density of 50 mA cm^−2^. (**E**) LSVs obtained for unmodified NiF (curve a), RuO_2_ NPs/NiF (curve b), NiO/NiF (curve c), Ni_2_P NPs/NiF (curve d), and Ni_2_P@NiO/NiF-40 (curve e) catalysts electrodes in 1.0 M KOH + urea-rich wastewater at a scan rate of 5 mV s^−1^. (**F**) LSVs obtained for Ni_2_P@NiO/NiF-40 catalyst electrode in 1.0 M KOH + urea-rich wastewater (curve a), and 1.0 M KOH + human urine sample (curve b) at a scan rate of 5 mV s^−1^.

The stability of the catalysts is an important factor to evaluate the EOU performance; thus, a longer durability of catalysts might deliver significant activity in real samples for practical application (wastewater treatment). We performed a chronopotentiometry measurement to investigate the stability of the as-synthesized Ni_2_P@NiO/NiF nanosheets electrode at a constant current density of 50 mA cm^−2^ for 24 h. The current density revealed a slight decrease at the first 1 h, which may be due to the activation of the catalyst. As the electrochemical reaction proceeded, the Ni_2_P@NiO/NiF nanosheet electrode showed a stable anodic current density profile due to the consumption of urea. After 12 h and 24 h of the electrochemical scan, the measured voltage values were 1.49 V and 1.47 V, retaining 98% of its initial chronopotentiometry response (Figure 6A; curve a). In addition, the LSV curves (Figure 6B) were obtained using an Ni_2_P@NiO/NiF nanosheet electrode before (curve a) and after 24 h (curve b) of the chronopotentiometry stability test, which showed that the catalyst has excellent stability. We further studied the extended durability or robustness of the as-synthesized Ni_2_P@NiO/NiF nanosheet catalyst using LSV measurements in 1.0 M KOH + 0.33 M urea at a scan rate of 5 mV s^−1^. The LSV profiles recorded for the freshly prepared, 1-, 3-, 5-, and 7-day stored Ni_2_P@NiO/NiF nanosheet-modified electrode (the Ni_2_P@NiO/NiF nanosheet-modified electrode was stored in Millipore water in ambient temperature under nitrogen atmosphere) displayed stable EOU responses (Figure 6C; curve a–e). The initial and 7-day stored Ni_2_P@NiO/NiF nanosheet-modified electrode showed the potential values of 1.36 V and 1.38 V at 50 mA cm^−2^ current density, respectively (only 20 mV shift), indicating long lasting stability and durability. Next, we performed a chronopotentiometry measurement to investigate the robustness of the Ni_2_P@NiO/NiF nanosheet electrode after 7 days at a constant current density of 50 mA cm^−2^ for 15 h (Figure 6D; curve a). The obtained result reveals a stable anodic current density profile with a 50 mA cm^−2^ at 1.39 V, indicating the longer durability of the catalyst. It is important to mention here that the longer durability of the Ni_2_P@NiO/NiF nanosheet catalyst at large current densities along with other catalysts can be applied for practical applications, i.e., for the EOU in urea-rich wastewater. As shown in Figure 6E, the EOU reaction was performed in 1.0 M KOH with urea-rich wastewater using the unmodified NiF (curve a), RuO_2_ NPs/NiF (curve b), Ni_2_P NPs/NiF (curve c), NiO NPs/NiF (curve d), and Ni_2_P@NiO/NiF-40 (curve e) catalyst electrodes. Among these electrodes, the Ni_2_P@NiO/NiF-40 catalyst electrode showed enhanced electro-oxidation of urea (50 mA cm^−2^ at 1.55 V) with a low-onset potential (1.42 V), indicating its excellent practicality for use in urea degradation for environmental safety and management applications. Finally, we compared the performance of the Ni_2_P@NiO/NiF-40 catalyst electrode toward EOU activity in urea-rich wastewater (curve a) and a human urine sample (curve b). We added 5 mL of human urine into a 1.0 M KOH solution and then tested it using the Ni_2_P@NiO/NiF-40 catalyst electrode at a scan rate of 5 mV s^−1^. The LSV results exhibit that a fairly similar onset potential is essential to achieve an identical current response in human urine. However, it has a marginally positive potential side in 1 M KOH + urea-rich wastewater (Figure 6F; curve a), which is mainly due to the effect of the complex of other organic components in human urine (Figure 6F; curve b). The higher activity as well as the long-term stability of the Ni_2_P@NiO/NiF-40 catalyst can be considered as a highly efficient candidate for the EOU process in fuel cell catalyst development and environmental safety applications.

## 4. Conclusions

We fabricated 3D wall-like Ni_2_P@NiO networked nanosheet hetero-structures on an Ni foam substrate via acid etching followed by a vapor-phase phosphating process. The morphological characterizations (SEM and TEM) exhibited that Ni_2_P nanoparticles were grown on the surface of the wall-like NiO nanosheets with respect to various phosphating process times. Notably, a 40 min phosphating process displayed the uniform growth of Ni_2_P nanoparticles on the surface as well as inside the pores via the Kirkendall effect. Among the developed catalyst materials, the 3D wall-like Ni_2_P@NiO networked nanosheet electrode showed an enhanced EOU reaction with a current response of 50 mA cm^−2^ at 1.35 V as well as a lower onset potential (1.31 V). The obtained enhanced electrocatalytic response of the wall-like Ni_2_P@NiO networked nanosheets offered a large active surface area and an amplified rate of mass transfer, which highly ascertained the synergism between the Ni_2_P nanoparticles and NiO nanosheets. The examination on the mechanism of the EOU on the wall-like Ni_2_P@NiO networked nanosheets reveals that the oxides in the components may be regularly transformed into corresponding oxyhydroxides, which play a pronounced role in EOU electrocatalysis. For practical applications, the wall-like Ni_2_P@NiO networked nanosheets showed a notable EOU response on urea-rich wastewater as well as human urine sample. Thus, the synthesized wall-like Ni_2_P@NiO nanosheet hetero-structured nanomaterial could be promising catalysts for the efficient electro-oxidation of urea in urea-rich wastewater remediation for energy and environmental safety applications.

## Data Availability

Not applicable.

## Supplementary Materials

The following supporting information can be downloaded at: https://www.mdpi.com/article/10.3390/nano12203633/s1, Figure S1: EDX and STEM analysis of Ni2P@NiO/NiF–40 catalyst; Figure S2: XRD of Ni2P@NiO catalyst prepared at 20, and 60 min phosphating time; Figure S3: XPS spectra for Ni 2p and O 1s of Ni(OH)2/NiF catalyst; Figure S4: LSVs of Ni2P@NiO/NF-40 catalyst electrode displaying OER and EOU profiles; Figure S5: Mass activity based EOU responses at Ni2P NPs/NiF, NiO/NiF, and Ni2P@NiO/NiF; Figure S6: CVs obtained at different scan rates (20 mV/s to 100 mV/s) of Ni2P@NiO/NF-40, NiO/NF, Ni2P/NF and NiF toward EOU; Table S1: Comparison of UOR performance of some representative reported Ni-based catalysts. References [33,63,64,65,66,67,68,69] were cited in the Supplementary Materials.
