# Supplementary material for: Ni2P Nanoparticle-Inserted Porous Layered NiO Hetero-Structured Nanosheets as a Durable Catalyst for the Electro-Oxidation of Urea
Source: Nanomaterials (Basel). 2022 Oct 17;12(20):3633. doi: 10.3390/nano12203633 (PMC9611741; doi:10.3390/nano12203633)
Supplement: Supplementary file 1 [file nanomaterials-12-03633-s001.zip › nanomaterials-1930157-supplementary.pdf]

Supplementary Materials

Ni_2P Nanoparticle-Inserted Porous Layered NiO Hetero-Structured Nanosheets as a Durable Catalyst for the Electro-Oxidation of Urea

Kun Ma [1], Hui Wang [2,*], Palanisamy Kannan [2,*] and Palaniappan Subramanian [3,*]

[1] Department of Internet, Jiaxing Vocational Technical College, Jiaxing 314001, China;
[2] College of Biological, Chemical Sciences and Engineering, Jiaxing University, Jiaxing 314001, China
[3] New Technologies-Research Center, University of West Bohemia, 30100 Plzeň, Czech Republic
* Correspondence: huiwang@zjxu.edu.cn (H.W.); ktpkannan@zjxu.edu.cn (P.K.); palans@ntc.zcu.cz (P.S.);
Tel.: +86-19857386580 (P.K.); Fax: +86-573-83643264 (P.K.)

LSV, EIS, and ECSA measurements.

LSV measurements were performed at a scan rate of 5 mV s^{-1} and corrected for *iR* compensation (100.0%). Next, EIS analysis was carried out for EOU by applying electrode frequency from 0.01 to 1,000,000 Hz at a amplitude of 5 mV and 1.34 V (RHE) potential was applied for EIS measurements. The ECSA of as-fabricated catalyst electrodes were accessed by double-layer capacitance (C_{dl}) that obtained through CV responses of non-Faradaic areas from -0.1 V to 0.0 V vs. Hg/HgO at scan rates from 20 mV s^{-1} to 100 mV s^{-1}. The catalytic current at -0.1 V and 0.0 V was used to obtain C_{dl} and ECSA based on given formula:

$$j = \nu \, . \, C_{dl}$$

$$\text{ECSA} = C_{dl} / (C_s \times A)$$

where j = double-layer charging current density, v = scan rate, C_s = specific capacitance of catalyst electrode (mF cm^{-2}), and A = area of working electrode (cm^2). C_s value of alkaline solution was referred as 40.0 mF cm^{-2}.

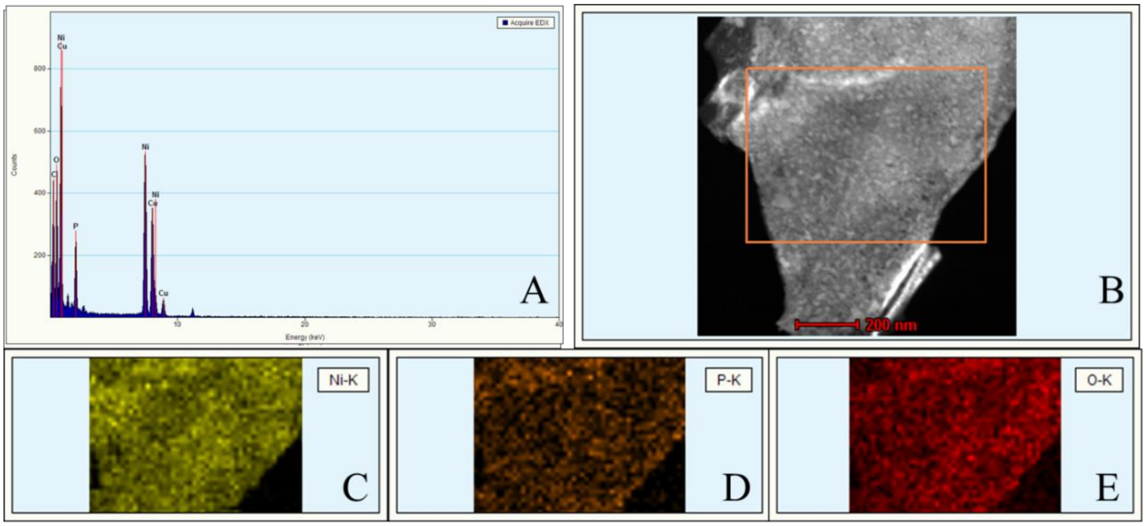

Figure S1. EDX analysis of Ni_2P@NiO/NiF-40 catalyst sample (A), and corresponding STEM and EELS elemental mappings of Ni, O, P elements (B-E).

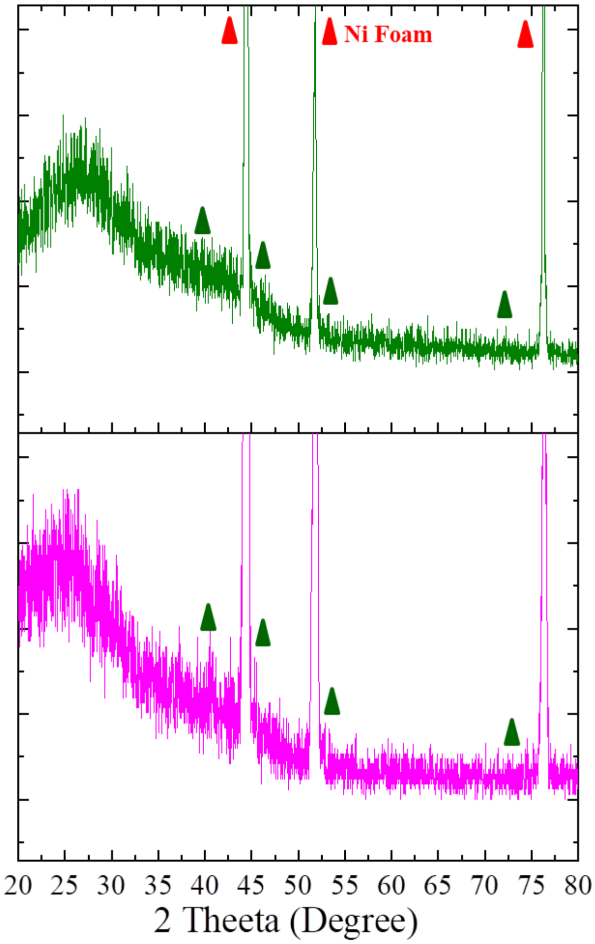

Figure S2. XRD pattern profiles of Ni_2P@NiO catalyst samples prepared at 20 min (top), and 60 min (bottom) phosphating time.

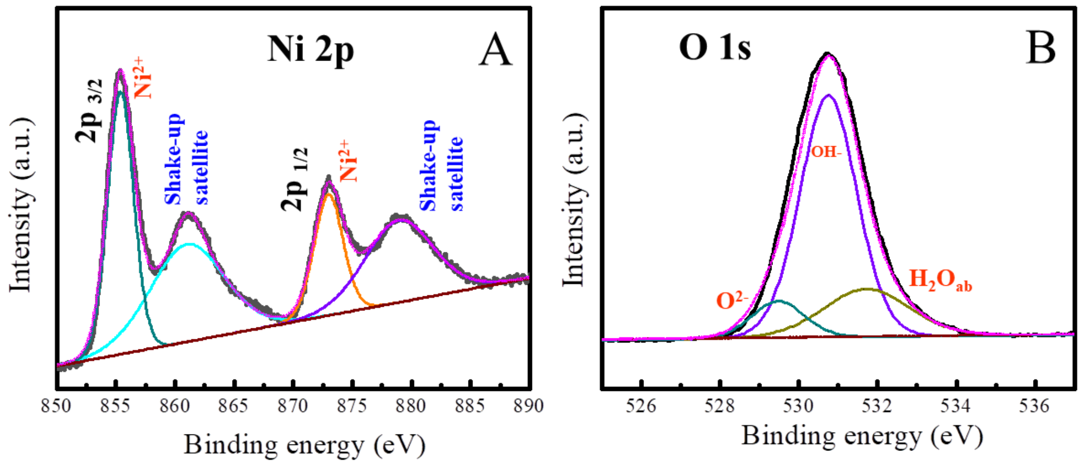

Figure S3. XPS spectra obtained for Ni 2p (A) and O 1s (B) of $Ni(OH)_2$/NiF catalyst sample.

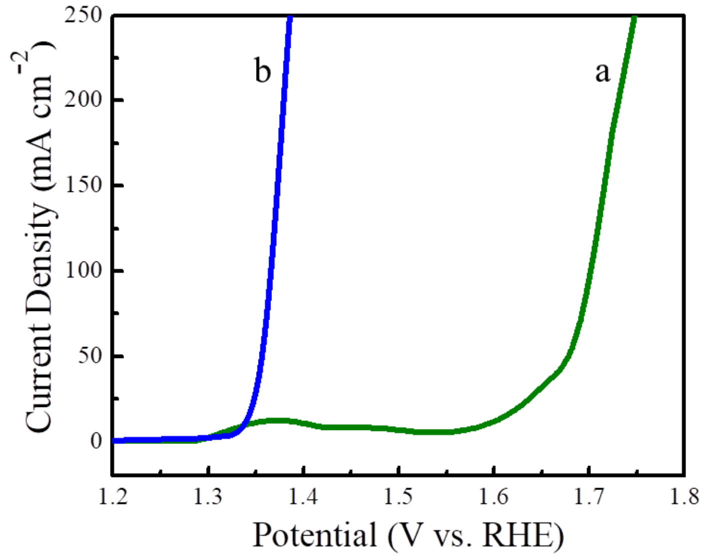

Figure S4. LSVs of Ni_2P@NiO/NF-40 catalyst electrode demonstrating the difference between OER (a) and EOU (b) profiles in 1 M KOH alone and 1.0M KOH + 0.33M urea at a scan rate of 5 mV s^{-1}.

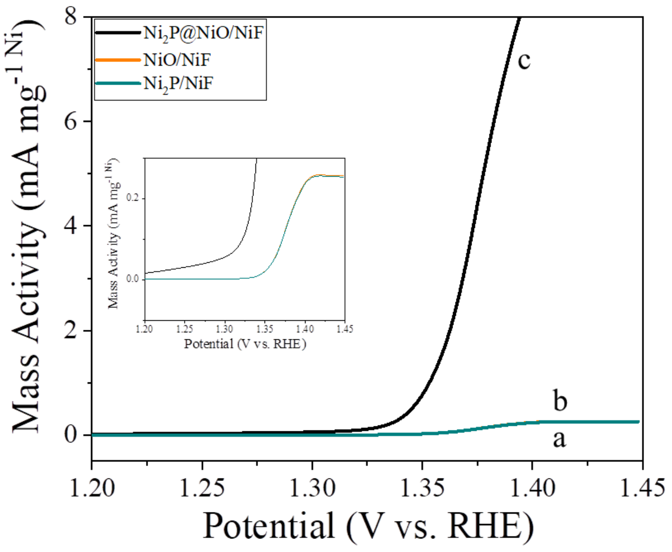

Figure S5. Mass activity based EOU responses obtained for Ni_2P NPs/NiF (line a), NiO/NiF (line b), and Ni_2P@NiO/NiF (curve c). Inset: Expanded view of Ni_2P NPs/NiF (line a), and NiO/NiF (line b).

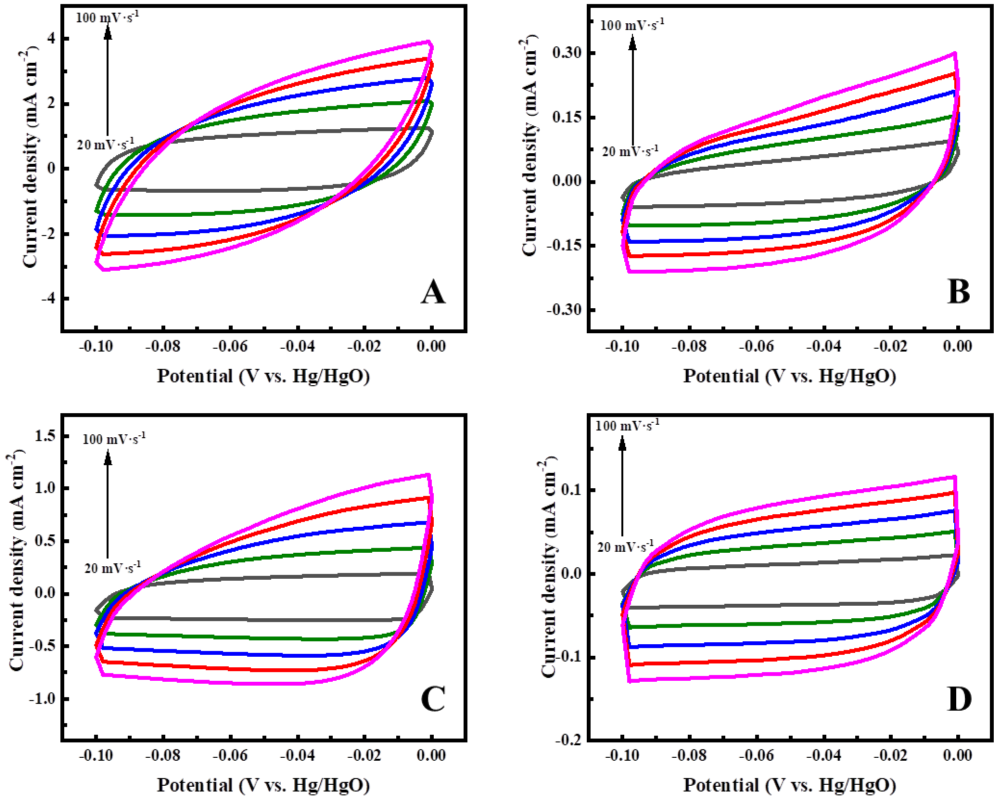

Figure S6. Cyclic voltammetry curves obtained at different scan rates (20 mV/s to 100 mV/s) of Ni_2P@NiO/NF-40 (a), NiO/NF (b), Ni_2P/NF (c) and NiF (d) for EOU reaction.

Table S1. Comparison of UOR performance of some representative reported Ni-based catalysts used for urea electrolysis.

Nanomaterials	Structure	Electrolyte	UOR Potential (V vs. RHE)	References
$Ni_2P/Fe_2P/NiF$	Nanoparticle	1 M KOH+0.5 M Urea	1.36 (10 mA cm^{-2}	[1]
Ni-Mo/NiF	Nanotube	1 M KOH+0.1 M Urea	1.36 (10 mA cm^{-2})	[2]
FQD/CoNi-LDH/NiF	Nanosheet	1 M KOH+0.5 M Urea	1.36 (10 mA cm^{-2})	[3]
Nickel terephthalate	MOF Nanosheet	1 M KOH+0.5 M Urea	1.381 (10 mA cm^{-2})	[4]
MoP@NiCo-LDH/NiF	Nanosheet	1 M KOH+0.5 M Urea	1.392 (100 mA cm^{-2})	[5]
Ni_3N/NiF	Nanosheet	1 M KOH+0.5 M Urea	1.406 (100 mA cm^{-2})	[6]
$Ni_2P/Ni_{0.96}S/NiF$	Microsphere	1 M KOH+0.5 M Urea	1.441 (100 mA cm^{-2})	[7]
Fe/Ni oxide	Nanorod	1 M KOH+0.33 M Urea	1.45 (30 mA cm^{-2})	[8]
Ni_2P@NiO/NIF	Nanosheet	1 M KOH+0.33 M Urea	1.35 (50 mA cm^{-2})	**This work**

References

1. Yan, L.; Sun, Y.; Hu, E.; Ning, J.; Zhong, Y.; Zhang, Z.; Hu, Y., Facile in-situ growth of Ni2P/Fe2P nanohybrids on Ni foam for highly efficient urea electrolysis. *Journal of Colloid and Interface Science* **2019,** 541, 279-286.
2. Zhang, J.-Y.; He, T.; Wang, M.; Qi, R.; Yan, Y.; Dong, Z.; Liu, H.; Wang, H.; Xia, B. Y., Energy-saving hydrogen production coupling urea oxidation over a bifunctional nickel-molybdenum nanotube array. *Nano Energy* **2019,** 60, 894-902.
3. Feng, Y.; Wang, X.; Huang, J.; Dong, P.; Ji, J.; Li, J.; Cao, L.; Feng, L.; Jin, P.; Wang, C., Decorating CoNi layered double hydroxides nanosheet arrays with fullerene quantum dot anchored on Ni foam for efficient electrocatalytic water splitting and urea electrolysis. *Chemical Engineering Journal* **2020,** 390, 124525.
4. Zheng, S.; Zheng, Y.; Xue, H.; Pang, H., Ultrathin nickel terephthalate nanosheet three-dimensional aggregates with disordered layers for highly efficient overall urea electrolysis. *Chemical Engineering Journal* **2020,** 395, 125166.
5. Wang, T.; Wu, H.; Feng, C.; Zhang, L.; Zhang, J., MoP@NiCo-LDH on nickel foam as bifunctional electrocatalyst for high efficiency water and urea–water electrolysis. *Journal of Materials Chemistry A* **2020,** 8, (35), 18106-18116.
6. Zhao, Z.; Zhao, J.; Wang, H.; Li, X.; Yang, L.; Zhao, Z.; Liu, X.; Liu, Y.; Liu, P.; Cai, Z., Porous flower-like nickel nitride as highly efficient bifunctional electrocatalysts for less energy-intensive hydrogen evolution and urea oxidation. *International Journal of Hydrogen Energy* **2020,** 45, (28), 14199-14207.

7. He, M.; Feng, C.; Liao, T.; Hu, S.; Wu, H.; Sun, Z., Low-Cost Ni2P/Ni0.96S Heterostructured Bifunctional Electrocatalyst toward Highly Efficient Overall Urea-Water Electrolysis. *ACS Applied Materials & Interfaces* **2020,** 12, (2), 2225-2233.

8. Gu, X.; Yang, D.; Liu, Z.; Wang, S.; Feng, L., Iron oxide promoted nickel/nickel oxide rough nanorods for efficient urea assisted water splitting. *Electrochimica Acta* **2020,** 353, 136516.
